# Visual Performance and Patient-Reported Visual Function with Multifocal, Monofocal, and Enhanced Monofocal Intraocular Lenses

**DOI:** 10.3390/jcm15218651

**Published:** 2026-11-06

**Authors:** Hadas Ben-Eli, Inbar Wunch, Riki Maroko, Tamar Winnett, Deborah Berrih-Elkouby, Arie L. Marcovich

**Affiliations:** 1 Department of Optometry and Vision Science, Jerusalem Multidisciplinary College, 37 Hanevi’im St., Jerusalem 9101001, Israel; inbar.wunch@gmail.com (I.W.); rikiko2311@gmail.com (R.M.); 99tamari99@gmail.com (T.W.); deborah.berrih87@gmail.com (D.B.-E.); 2 Department of Ophthalmology, Hadassah-Medical Centre and the Faculty of Medicine, the Hebrew University of Jerusalem, Jerusalem 9112001, Israel; 3 Department of Ophthalmology, Kaplan Medical Center, Rehovot 7661041, Israel; arie.marcovich@gmail.com; 4 Faculty of Medicine, Hebrew University of Jerusalem, Jerusalem 9112102, Israel

**Keywords:** cataract, intraocular lens, monofocal, multifocal, visual acuity, contrast sensitivity

## Abstract

**Objective:** The aim of the study was to compare visual performance and patient-reported visual function among the enhanced monofocal TECNIS Eyhance, conventional monofocal Sensar, and multifocal Synergy intraocular lenses (IOLs) (Johnson & Johnson Surgical Vision, Inc., Santa Ana, CA, USA). **Methods:** This prospective comparative observational study included patients assessed after cataract surgery with Eyhance, Sensar, or Synergy implantation. Uncorrected and corrected visual acuity were measured at distance, intermediate, and near, and contrast sensitivity was assessed using the Pelli-Robson chart. Participants completed the validated Visual Function Index (VF-14) and three nonvalidated exploratory questions addressing unaided intermediate vision. Eye-level outcomes were analyzed using linear mixed-effects models accounting for fellow-eye correlation and adjusted for age and preoperative spherical equivalent. The VF-14 was analyzed once per participant. **Results:** Fifty-two participants were enrolled, of whom 51 contributed 84 eligible eyes to the analysis: 40 Eyhance, 26 Synergy, and 18 Sensar eyes. Among the 51 analyzed participants, 31 (60.8%) were women. Mean age of participants was 69.98 ± 7.90 years, with an age range of 48–85 years. Adjusted uncorrected distance visual acuity did not differ among the IOL groups (*p* = 0.994). Synergy provided better uncorrected intermediate and near visual acuity than Eyhance and Sensar (overall *p* < 0.001 for both), whereas Eyhance and Sensar did not differ. Corrected visual acuity did not differ significantly among the groups at any distance. Contrast sensitivity differed at distance (*p* = 0.007) and near (*p* = 0.011); Synergy had lower values than both monofocal-type IOLs, whereas Eyhance and Sensar were comparable. Adjusted VF-14 scores did not differ among the groups (*p* = 0.451). Exploratory intermediate-vision responses favored Synergy but demonstrated a pronounced ceiling effect. **Conclusions:** All three IOLs provided comparable uncorrected distance visual acuity. Synergy was associated with better unaided intermediate and near visual acuity but lower contrast sensitivity, whereas Eyhance showed no objective advantage over Sensar. Overall patient-reported visual function did not differ among the groups, and findings from the additional intermediate-vision questions were exploratory.

## 1. Introduction

Cataract remains a leading cause of visual impairment worldwide, affecting approximately 30% of individuals aged 60–69 years and 64% of those aged ≥70 years [1]. The growing volume of cataract surgery has shifted its goals beyond restoring distance visual acuity toward providing functional vision across multiple distances [2]. Consequently, IOL selection increasingly considers patients’ visual demands, lifestyles, expectations regarding spectacle independence, and tolerance of optical side effects [3]. Current IOL development therefore aims to improve intermediate and near vision while preserving distance vision, contrast sensitivity, and visual quality [4].

The TECNIS Eyhance IOL (ICB00; Johnson & Johnson Surgical Vision, Santa Ana, CA, USA) is an enhanced monofocal IOL designed to improve intermediate vision while maintaining the optical characteristics of a conventional monofocal lens [5,6]. Randomized and observational studies have reported better intermediate visual acuity with Eyhance than with conventional monofocal IOLs, with comparable distance acuity, contrast sensitivity, and photic phenomena [5,7]. Large meta-analyses of randomized trials and nine comparative studies similarly demonstrated improved intermediate and near visual acuity and greater intermediate spectacle independence without clinically meaningful differences in distance vision, contrast sensitivity, or photic phenomena [7,8].

Enhanced monofocal IOLs, however, generally provide a narrower range of vision than EDOF and multifocal lenses. Non-diffractive EDOF lenses, including TECNIS PureSee and AcrySof IQ Vivity, have demonstrated better intermediate and near visual acuity and broader defocus ranges than Eyhance, while maintaining comparable distance vision and contrast sensitivity [9,10]. Diffractive multifocal IOLs can further improve near vision but may increase halos, glare, and starbursts and reduce contrast sensitivity, particularly under mesopic or glare conditions [11,12,13]. These trade-offs vary according to optical design; for example, diffractive bifocal IOLs may provide better near acuity, whereas refractive multifocal designs may better preserve contrast sensitivity [14]. Accordingly, both objective visual performance and patient-reported outcomes are important when selecting an IOL.

Independent head-to-head comparisons of Eyhance, Sensar, and Synergy IOLs (Johnson & Johnson Surgical Vision, Inc., Santa Ana, CA, USA) within a single clinical cohort remain limited. Comparing these platforms using both objective visual measures and patient-reported outcomes may clarify the trade-offs between range of vision and contrast sensitivity and thereby support individualized IOL selection. Accordingly, this study compared uncorrected and corrected visual acuity at distance, intermediate, and near; distance and near contrast sensitivity; and patient-reported visual function among patients implanted with the enhanced monofocal TECNIS Eyhance, conventional monofocal Sensar, or multifocal TECNIS Synergy IOL.

## 2. Materials and Methods

### 2.1. Participants

This prospective, comparative observational study enrolled patients after cataract surgery. The study protocol did not determine or modify the surgical procedure or IOL allocation. The study was approved by the IRB committee of the Jerusalem Multidisciplinary College (study #: 155. 6 June 2021). Participants received oral and written information about the study aims, methods and signed informed consent form prior to enrollment. All data were anonymized and coded for both clinical examination and questionnaire for statistical analysis.

Participants had undergone cataract surgery in one or both eyes by the same surgeon (A.M.) using a standardized surgical approach and were evaluated between 1 and 12 months postoperatively. They were classified according to the implanted IOL: Eyhance enhanced monofocal, Sensar monofocal, or Synergy multifocal. Patients were excluded if they had clinically significant posterior capsule opacification (PCO) requiring Nd:YAG laser capsulotomy or any intraoperative or postoperative ocular complication that could affect visual outcomes. Clinically significant posterior capsule opacification requiring treatment was assessed before visual-function testing. One eye with untreated PCO requiring Nd:YAG laser capsulotomy was not examined and was excluded from the analysis. A second participant was assessed approximately one month after Nd:YAG capsulotomy, once the treated eye was considered clinically stable. One additional eye had mild PCO that did not require treatment and remained eligible for analysis. Thus, no eye with untreated, clinically significant PCO was included. Preoperative cataract type and severity and the presence of pterygium were obtained from the medical records. Additional exclusion criteria were corneal astigmatism exceeding 1.00 D against the rule or 1.50 D with the rule requiring toric IOL implantation; ocular or systemic disease that could impair vision; medications affecting visual acuity or the ocular surface; severe dry-eye disease; amblyopia, defined as corrected visual acuity worse than 6/12 (0.30 logMAR); and failure to provide informed consent.

### 2.2. Recruitment

The study was conducted at a private ophthalmology clinic (A.M.) during routine postoperative checkups. Patients who had cataract surgery with the same surgeon (A.M.) and were implanted with the Eyhance, Sensar or Synergy IOLs in the past year prior to the enrollment were examined at least one month after the surgery. During this examination, optometrists (T.W., R.M., I.W., D.B.) approached the patients to participate in the study. Participation in the study was voluntary and did not affect the patients’ care or treatment. The optometrists who performed the standardized visual-function assessments were masked to the implanted IOL, whereas the operating surgeon was aware of the IOL type. The IOL information was not disclosed to the optometrists before or during testing, and they completed the assessments before the participant underwent the surgeon’s clinical examination. The optometric evaluation included visual acuity at distance, intermediate, and near, subjective refraction, contrast sensitivity and pupil-size measurement. Also, the optometrists did not review the surgical records or access information identifying the implanted IOL before completing the study measurements.

### 2.3. IOL Selection

IOL allocation was not randomized and occurred before study enrollment as part of routine clinical care. Lens selection was based on shared decision-making between the patient and surgeon, considering ocular suitability, visual priorities, lifestyle, desired spectacle independence, and acceptance of potential optical trade-offs. Patients preferring conventional monofocal correction generally received Sensar. Eyhance was offered to patients seeking a potential improvement in intermediate vision while preferring a monofocal-type optical profile. Synergy was selected by patients prioritizing a broader range of unaided vision and willing to accept the potential optical effects associated with a diffractive multifocal design.

### 2.4. Procedure

After eligibility was confirmed, each participant was examined by an optometrist who was masked to the implanted IOL and performed all standardized visual-function assessments. These included uncorrected distance visual acuity using a logMAR chart at 6 m; intermediate and near visual acuity using a Jaeger test card at 70 and 40 cm, respectively [15]; subjective refraction; best-corrected visual acuity at distance, intermediate, and near; contrast sensitivity at distance (1 m) and near (40 cm) using the Pelli–Robson chart (Precision Vision, Inc., Woodstock, IL, USA); and pupil-size measurement using a ruler. After the optometric assessment, the participant underwent a clinical examination by the operating surgeon, who was aware of the implanted IOL.

Participants completed the validated 14-item Visual Function Index (VF-14) to assess vision-related functional difficulty after cataract surgery [16]. Each item was scored from 0 to 4, and the total score was calculated by dividing the sum of the item scores by the number of completed items and multiplying by 25. Scores ranged from 0 to 100, with higher scores indicating less difficulty with vision-dependent activities. The questionnaire was translated from English into Hebrew and back-translated into English, with the translation reviewed by a bilingual speaker.

Because the VF-14 does not specifically assess several intermediate-vision tasks relevant to contemporary IOLs, participants were subsequently contacted by telephone and asked three study-specific questions concerning unaided difficulty with: (1) reading small print, such as medication or food labels; (2) reading a book or newspaper; and (3) performing intermediate-distance activities, including computer use, reading musical notes, playing card games, and cooking. These questions were not part of the validated VF-14, were not included in its total score, and were analyzed separately as exploratory outcomes.

Preoperative medical and ocular history, refraction, and distance and near best-corrected visual acuity were obtained from the participants’ medical records.

### 2.5. Statistical Analysis

Continuous variables are presented as means and standard deviations, and categorical variables as counts and percentages. Preoperative spherical equivalent was calculated as sphere plus one-half cylinder. Because some participants contributed both eyes, eye-level outcomes were analyzed using linear mixed-effects models with IOL type as a fixed effect and a participant-specific random intercept to account for within-participant correlation. Models were adjusted for age and preoperative spherical equivalent; models of postoperative uncorrected and corrected distance visual acuity were additionally adjusted for preoperative distance visual acuity. Adjusted marginal means, between-group differences, and 95% confidence intervals were reported. Pairwise comparisons were corrected for multiple testing using the Holm method.

The VF-14 was analyzed once per participant using multivariable linear regression adjusted for age and participant-level mean preoperative spherical equivalent. The three study-specific intermediate-vision questions were analyzed separately from the VF-14 as exploratory ordinal outcomes. The intermediate-activity item was evaluated using the same participant-level covariate-adjusted model; adjusted marginal means, 95% confidence intervals, and the overall Wald F-test *p* value were reported. Given the small sample and ceiling effect, this analysis was considered hypothesis-generating. Complete-case analysis was performed without imputation, and the sample size for each analysis was reported. Effect estimates and 95% confidence intervals were used to assess the magnitude and precision of the observed associations. All tests were two-sided, and *p* < 0.05 was considered statistically significant. Data management and descriptive analyses were performed using IBM SPSS Statistics version 27.0 (IBM SPSS Statistics, Version 27.0, Chicago; Armonk, NY, USA: IBM Corp.). Linear mixed-effects models, adjusted marginal means, and Holm-adjusted pairwise comparisons were performed using Python version 3.12 (Python Software Foundation, Wilmington, DE, USA).

## 3. Results

### 3.1. Participant and Baseline Characteristics

Fifty-two participants were enrolled, of whom 51 contributed 84 eligible eyes to the analysis. Among the analyzed participants, 31 (60.8%) were women, 33 contributed two eligible eyes, and 18 contributed one eligible eye. Mean age of study participants was 69.98 ± 7.90 years and ranged between 48–85 years. Of the included eyes, 40 were implanted with an Eyhance IOL, 26 with a Synergy IOL, and 18 with a Sensar IOL. IOL selection occurred before study enrollment through routine shared decision-making between the patient and surgeon; no study-specific randomization or treatment assignment was performed. Baseline characteristics are presented in Table 1. Preoperative spherical equivalent differed significantly among the IOL groups (*p* = 0.007), whereas the difference in age did not reach statistical significance (*p* = 0.059). Age and preoperative spherical equivalent were therefore included as covariates in the adjusted analyses.

### 3.2. Visual Acuity Outcomes

After adjustment for age, preoperative spherical equivalent, and within-participant correlation, uncorrected distance visual acuity (UDVA) did not differ among the three IOL groups (*p* = 0.994). In contrast, significant overall group differences were observed for uncorrected intermediate visual acuity (UIVA) and uncorrected near visual acuity (UNVA; both *p* < 0.001). Synergy provided better UIVA than Eyhance (*p* < 0.001) and Sensar (*p* = 0.002), and better UNVA than both Eyhance and Sensar (both *p* < 0.001). Eyhance and Sensar did not differ significantly in UIVA (*p* = 0.973) or UNVA (*p* = 0.946).

No significant overall group differences were observed for corrected distance visual acuity (CDVA; *p* = 0.108), corrected intermediate visual acuity (CIVA; *p* = 0.206), or corrected near visual acuity (CNVA; *p* = 0.201). None of the Holm-adjusted pairwise comparisons for corrected visual acuity were statistically significant (Table 2).

### 3.3. Contrast Sensitivity

After adjustment for age, preoperative spherical equivalent, and within-participant correlation, contrast sensitivity differed significantly among the IOL groups at distance (overall *p* = 0.007) and near (overall *p* = 0.011; Table 3). Adjusted distance contrast sensitivity was lower with Synergy than with Eyhance and Sensar (Holm-adjusted *p* = 0.013 for both comparisons), whereas Eyhance and Sensar did not differ significantly (*p* = 0.511).

Similarly, adjusted near contrast sensitivity was lower with Synergy than with Eyhance (*p* = 0.008) and Sensar (*p* = 0.046). Eyhance and Sensar showed comparable near contrast sensitivity (*p* = 0.952).

### 3.4. Patient-Reported Visual Function and Satisfaction

**Figure 1:**
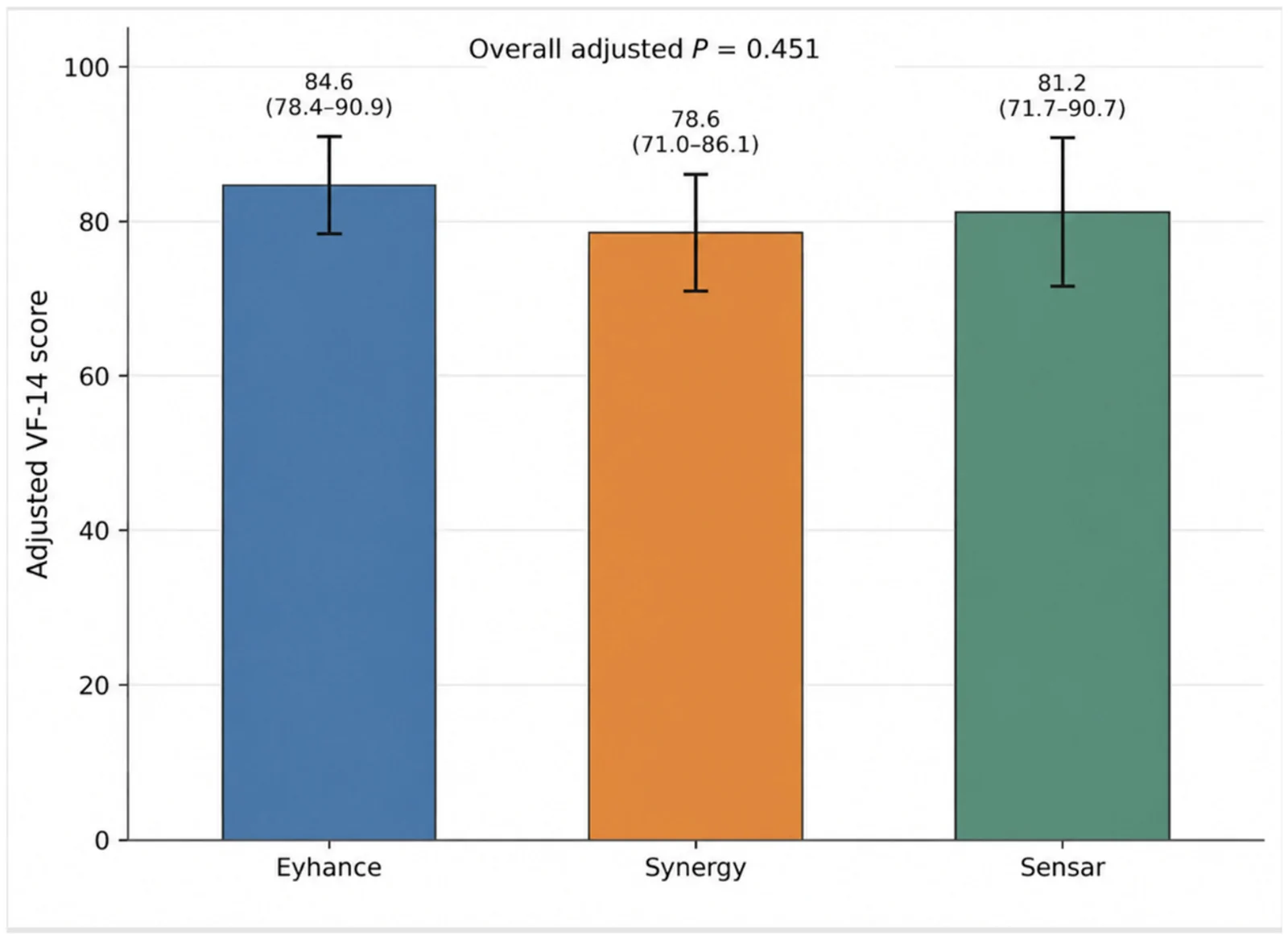
**Adjusted VF-14 scores according to intraocular lens type**. Estimated marginal mean VF-14 scores are presented for participants implanted with Eyhance, Synergy, or Sensar intraocular lenses. Estimates were obtained from a participant-level multivariable linear regression model adjusted for age and participant mean preoperative spherical equivalent. Error bars represent 95% confidence intervals. The adjusted VF-14 score did not differ significantly among the IOL groups (overall *p* = 0.451). The analysis included 48 participants with complete questionnaire and covariate data. VF-14, 14-item Visual Function Index; IOL, intraocular lens; CI, confidence interval.

The VF-14 was analyzed once per participant. After adjustment for age and participant-level mean preoperative spherical equivalent, VF-14 scores did not differ significantly among the IOL groups (*p* = 0.451). The adjusted mean scores were 84.65 for Eyhance, 78.55 for Synergy, and 81.20 for Sensar (Figure 1). No Holm-adjusted pairwise comparison was statistically significant.

**Figure 2:**
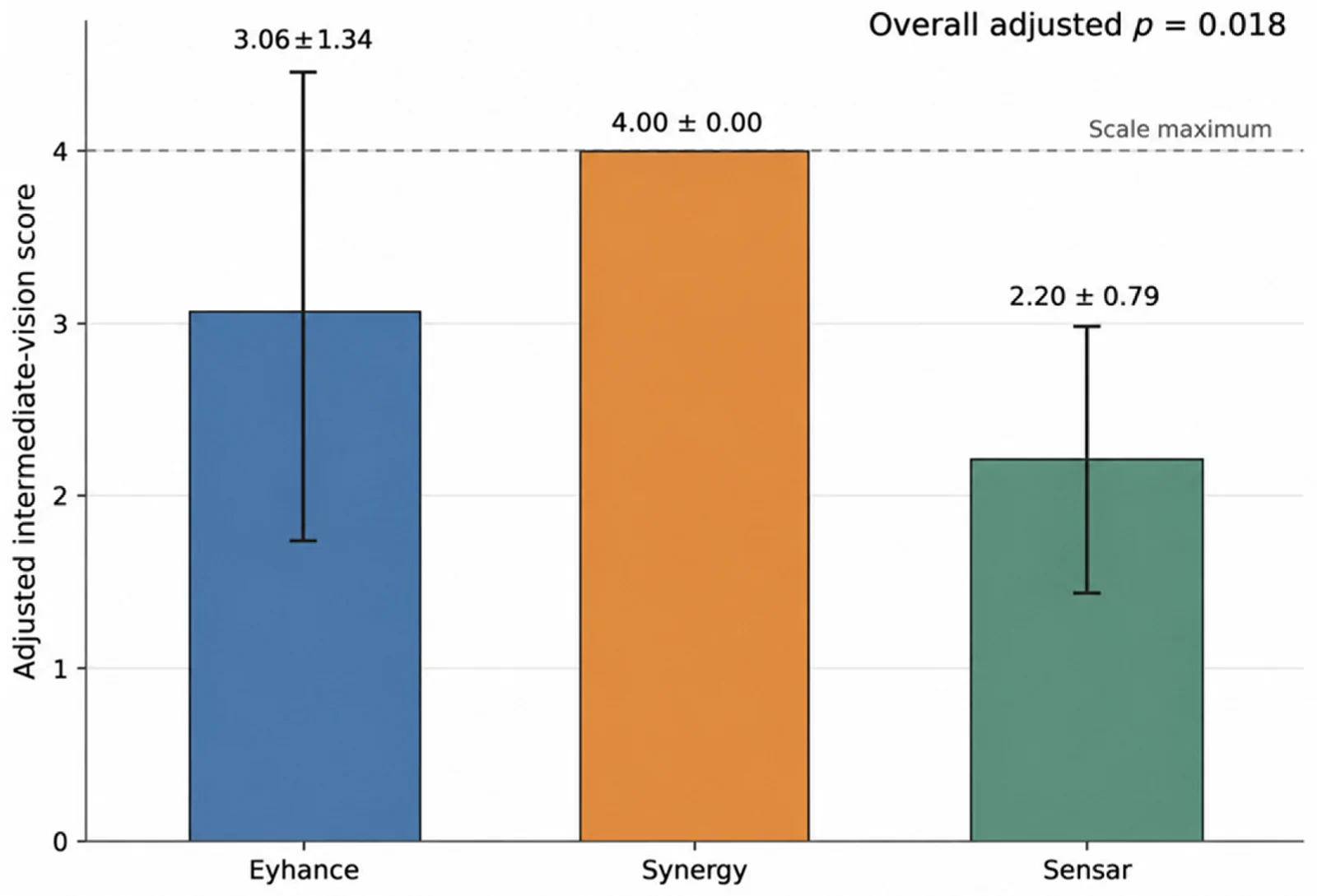
**Exploratory responses to study-specific questions on unaided intermediate visual function.** Observed mean response scores are presented for participants implanted with Eyhance, Synergy, or Sensar intraocular lenses (IOLs). Higher scores indicate less difficulty with unaided intermediate-distance activities. The overall group difference was evaluated using participant-level multivariable linear regression adjusted for age and participant-level mean preoperative spherical equivalent (overall adjusted *p* = 0.018; Wald F test). All Synergy participants selected the maximum response, indicating a pronounced ceiling effect. This nonvalidated item was analyzed separately from the VF-14 as an exploratory outcome. Error bars represent standard deviations.

Figure 2 presents responses to the exploratory study-specific ordinal item assessing unaided intermediate-distance activities. The observed mean ± SD scores were 3.06 ± 1.34 for Eyhance, 4.00 ± 0.00 for Synergy and 2.20 ± 0.79 for Sensar. All Synergy participants reported the maximum score, indicating a pronounced ceiling effect. The overall group difference remained significant in an exploratory analysis adjusted for age and participant-level mean preoperative spherical equivalent (*p* = 0.018).

## 4. Discussion

### 4.1. Principal Findings

This study compared visual performance and patient-reported outcomes after cataract surgery with the enhanced monofocal Eyhance, conventional monofocal Sensar, and diffractive multifocal Synergy IOLs. After adjustment for age, preoperative spherical equivalent, and within-participant correlation, uncorrected distance visual acuity was comparable among the three IOL groups. Synergy provided better uncorrected intermediate and near visual acuity than Eyhance and Sensar, whereas Eyhance did not demonstrate an objective intermediate- or near-vision advantage over Sensar. Corrected visual acuity did not differ significantly among the groups at any testing distance.

Eyhance and Sensar provided comparable contrast sensitivity at distance and near. Synergy had lower adjusted contrast sensitivity than both monofocal-type IOLs at both testing distances. However, overall VF-14 scores did not differ significantly among the groups after participant-level analysis and adjustment for age and preoperative spherical equivalent. Together, these findings demonstrate a trade-off between the broader range of unaided vision provided by Synergy and the higher contrast sensitivity observed with the monofocal-type IOLs.

### 4.2. Distance, Intermediate, and Near Visual Acuity

Adjusted uncorrected distance visual acuity did not differ among the three IOL groups. Corrected distance visual acuity was also comparable after adjustment for age, preoperative spherical equivalent, preoperative distance visual acuity, and within-participant correlation. These findings are consistent with those of Cinar et al. [17], who reported comparable corrected and uncorrected distance visual acuity after implantation of Eyhance and conventional monofocal IOLs.

Synergy provided better adjusted uncorrected intermediate and near visual acuity than both Eyhance and Sensar. This finding is consistent with the optical design of Synergy, which is intended to provide an extended range of vision from distance to near [18,19]. In contrast, corrected intermediate and near visual acuity did not differ significantly among the groups, suggesting that the principal advantage of Synergy was in unaided visual performance.

Eyhance did not demonstrate significantly better uncorrected intermediate or near visual acuity than Sensar in our cohort. Previous studies and meta-analyses have generally reported modest improvements in intermediate vision with Eyhance compared with conventional monofocal IOLs [5,6,17,20,21,22,23]. However, this advantage has not been consistent across all comparisons: a recent study found comparable intermediate visual acuity with Eyhance and Clareon [24], and other studies reported no difference in near visual acuity or contrast sensitivity despite better intermediate outcomes with Eyhance [25,26,27]. The spherical optical design of Sensar AR40e may retain positive spherical aberration and thereby broaden the depth of focus, potentially reducing the relative advantage of Eyhance; however, this explanation remains speculative because spherical aberration and defocus curves were not measured in our study [28]. Eyhance uses a modified anterior aspheric surface with a continuous central power gradient intended to extend functional vision while preserving distance image quality, but its measurable benefit may depend on the comparator lens and testing conditions [5,26].

Comparative studies have also shown that IOLs with a broader extended-depth-of-focus profile may provide better intermediate or near performance than Eyhance. Comparisons between Eyhance and the TECNIS Symfony platform found similar distance outcomes but generally better near performance with Symfony [23,29]. More recently, Son et al. reported that both Eyhance and the non-diffractive PureSee IOL provided excellent distance vision and refractive stability, whereas PureSee provided better intermediate and near visual performance [9]. These findings support the concept that enhanced monofocal IOLs may extend functional vision modestly but generally provide a narrower range than EDOF or multifocal designs.

Rocha et al. compared spherical and aspheric monofocal IOLs, including the Sensar AR40, and found comparable distance visual acuity but differences in distance-corrected intermediate and near visual acuity among the evaluated designs [30]. Such findings further indicate that visual performance depends on the specific optical characteristics of the IOL and cannot be inferred solely from its broad classification as monofocal, enhanced monofocal, or multifocal.

### 4.3. Contrast Sensitivity

After adjustment for age, preoperative spherical equivalent, and within-participant correlation, Eyhance and Sensar demonstrated comparable contrast sensitivity at distance and near. Thus, the enhanced monofocal design of Eyhance demonstrated similar contrast sensitivity to the conventional monofocal Sensar IOL but did not provide a preferred contrast-sensitivity advantage over it. This finding is consistent with Unsal and Sabur, who found no difference in photopic contrast sensitivity across spatial frequencies between Eyhance and a conventional TECNIS monofocal IOL [20].

Synergy demonstrated lower adjusted contrast sensitivity than both Eyhance and Sensar at distance and near. This finding may be related to its diffractive optical design, which distributes incoming light across different focal ranges and may consequently reduce retinal-image contrast [31]. By comparison, Eyhance uses a modified aspheric anterior surface and continuous central power gradient to extend the range of vision without dividing light into distinct foci [6].

Kim et al. similarly reported lower contrast sensitivity with a multifocal IOL than with a monofocal IOL across the tested spatial frequencies [32]. However, that study evaluated different IOL models, limiting direct comparison with the present results. Moreover, although the contrast-sensitivity differences in our study were statistically significant, their clinical importance remains uncertain. The absolute differences were modest, no threshold for a clinically meaningful difference was prespecified, and functional performance under mesopic, glare, or other low-contrast conditions was not evaluated. The results should therefore not be interpreted as demonstrating a clinically perceptible disadvantage of Synergy.

### 4.4. Patient-Reported Outcomes

The adjusted VF-14 score did not differ significantly among the IOL groups. This finding suggests that the objective differences in unaided visual range and contrast sensitivity did not translate into clearly distinguishable differences in general vision-related functioning within this cohort. The VF-14, however, was developed to assess general visual disability and may not adequately capture several intermediate-distance tasks that are relevant to contemporary refractive IOLs.

The three study-specific intermediate-vision questions favored Synergy. This finding is broadly consistent with the objective intermediate-vision advantage observed with this IOL and with previous reports of greater satisfaction with multifocal than monofocal IOLs for some visual tasks [33]. Wilkins et al., in a randomized comparison of a diffractive multifocal IOL and a monofocal IOL targeted for monovision, found no significant difference in overall satisfaction, although task-specific visual outcomes differed between the groups [34].

Nevertheless, the three additional questions were not part of the validated VF-14 and were not psychometrically validated. Furthermore, all Synergy participants provided the maximum response, indicating a pronounced ceiling effect. These results should therefore be considered exploratory and hypothesis-generating rather than evidence of a definitive difference in patient satisfaction.

### 4.5. Clinical Implications

The findings demonstrate that IOL selection involves a balance between unaided visual range and contrast sensitivity. Synergy was associated with better unaided intermediate and near visual acuity but lower contrast sensitivity. Eyhance and Sensar provided similar intermediate and near visual acuity and comparable contrast sensitivity. Thus, the present data do not support an objective intermediate-vision or contrast-sensitivity advantage of Eyhance over Sensar.

These findings may support individualized IOL selection by helping clinicians align the expected visual range and contrast sensitivity with each patient’s visual priorities. Because IOL allocation reflected routine clinical counseling, clinical suitability, and patient preferences rather than randomization, the results represent real-world associations. Halos, glare, dysphotopsia, spectacle independence, and night-driving performance [31] were beyond the scope of this study.

### 4.6. Strengths and Limitations

A strength of this study is the direct comparison of three clinically distinct IOL strategies using visual acuity at multiple distances, contrast sensitivity, and patient-reported visual function. The study also provides independent, non-industry-funded, real-world evidence reflecting routine IOL selection and postoperative care. All surgeries were performed by the same surgeon, which reduced variability related to surgical technique, and optometric testing was completed by examiners who were masked to the implanted IOL before the surgeon performed the clinical examination.

Several limitations should be considered. First, this was an observational study with nonrandomized IOL allocation. Patients receiving the three IOL types may have differed in visual priorities, lifestyle, expectations regarding spectacle independence, tolerance of potential optical effects, and measured or unmeasured clinical characteristics. The groups also differed in preoperative spherical equivalent, and age varied across groups. Although the analyses adjusted for age and preoperative spherical equivalent, residual confounding and confounding by indication cannot be excluded.

Second, the sample was modest and unevenly distributed among the IOL groups, particularly for Sensar. The complete cohort included 84 eyes from 51 participants, and the adjusted eye-level models included 81 eyes from 50 participants with complete covariate data. Fellow-eye correlation was addressed using participant-specific random-intercept models. Nevertheless, the effective number of independent participants remained limited. The original sample-size calculation was based on a two-group comparison and did not account for the final three-group design or correlation between fellow eyes. The study may therefore have had insufficient power to detect small differences, and nonsignificant findings should be interpreted as an absence of demonstrated differences rather than evidence of equivalence.

Third, retinal and ocular-surface factors that may influence contrast sensitivity were not comprehensively quantified. Although Synergy participants were younger, which would generally favor better contrast sensitivity, they showed lower values; thus, age is unlikely to explain this difference, particularly because the analyses were age-adjusted. Mesopic and glare conditions were not assessed, limiting interpretation of the clinical significance.

Fourth, clinically significant PCO was excluded, although mild PCO and unmeasured surgical characteristics may have had a limited effect on visual outcomes.

Fifth, optometrists were masked to IOL type during visual testing, although masking effectiveness was not formally assessed.

Finally, the VF-14 was analyzed once per participant and therefore avoided treating duplicated questionnaire responses as independent observations. However, one participant with different included IOL types could not be assigned to a single questionnaire group, and the adjusted VF-14 analysis was limited to participants with complete covariate data. In addition, the three study-specific intermediate-vision questions were not validated and demonstrated a pronounced ceiling effect. Their findings should therefore be interpreted cautiously.

## 5. Conclusions

In this observational real-world cohort, distance visual acuity did not differ significantly among the three IOL groups. Synergy was associated with better unaided intermediate and near visual acuity but lower contrast sensitivity. Eyhance did not demonstrate an objective advantage over Sensar in intermediate or near visual acuity or contrast sensitivity. Overall VF-14 scores did not differ significantly among the groups, while findings from the nonvalidated intermediate-vision questions were exploratory. These results may inform individualized IOL selection based on patients’ visual priorities but should be interpreted cautiously because of the nonrandomized allocation, modest sample size, and potential residual confounding.

## Figures and Tables

**Table 1:** Baseline characteristics of the study participants.

Characteristics	Eyhance	Synergy	Sensar	*p* Value
**Eyes/contributing participants**	40/25	26/17	18/10	*NA*
**Age, years, mean ± SD (participant level)**	69.71 ± 6.79	67.00 ± 9.05	74.72 ± 7.00	0.059 ^a^
**Age range (years)**	55–82	48–85	57–82	*NA*
**Women, n (participant level)**	11	14	6	0.06 ^b^
**Pre-operative distance VA (LogMAR)** **Mean ± SD**	0.31 ± 0.17	0.29 ± 0.15	0.32 ± 0.19	0.08 ^a^
**Pre-operative SE (D)** **Mean ± SD**	−0.44 ± 2.39	−0.50 ± 3.19	0.24 ± 2.90	0.007 ^a^

Age and sex are summarized at the participant level among participants contributing at least one eye to each IOL group; one participant contributed one eye to two different IOL groups and is therefore represented in both relevant columns. Ocular characteristics are summarized at the eye level. IOL, intraocular lens. ^a^ Tested by Kruskal–Wallis test. ^b^ Tested by Chi-Square test. VA = Visual Acuity, SD = standard deviation, SE = spherical equivalent, D = Diopter.

**Table 2:** Adjusted Visual Acuity Outcomes and Pairwise Comparisons Among the Three Intraocular Lens Groups.

Outcome	Group or Comparison	Adjusted Mean or Difference	95% CI	*p* Value
**UDVA**	Eyhance	0.062	0.033 to 0.090	Overall: 0.994
	Synergy	0.063	0.027 to 0.098	—
	Sensar	0.060	0.014 to 0.105	—
	Eyhance - Synergy	−0.001	−0.047 to 0.044	1.000
	Eyhance - Sensar	0.002	−0.052 to 0.056	1.000
	Synergy - Sensar	0.003	−0.057 to 0.063	1.000
**UIVA**	Eyhance	0.533	0.466 to 0.601	Overall: <0.001
	Synergy	0.297	0.215 to 0.380	—
	Sensar	0.531	0.424 to 0.638	—
	Eyhance - Synergy	0.236	0.131 to 0.341	<0.001
	Eyhance - Sensar	0.002	−0.125 to 0.129	0.973
	Synergy - Sensar	−0.234	−0.373 to −0.094	0.002
**UNVA**	Eyhance	0.547	0.453 to 0.641	Overall: <0.001
	Synergy	0.123	0.009 to 0.237	—
	Sensar	0.540	0.390 to 0.691	—
	Eyhance - Synergy	0.423	0.279 to 0.568	<0.001
	Eyhance - Sensar	0.006	−0.172 to 0.185	0.946
	Synergy - Sensar	−0.417	−0.612 to −0.223	<0.001
**CDVA**	Eyhance	0.015	−0.000 to 0.030	Overall: 0.108
	Synergy	0.011	−0.008 to 0.029	—
	Sensar	0.043	0.019 to 0.067	—
	Eyhance - Synergy	0.004	−0.020 to 0.028	0.741
	Eyhance - Sensar	−0.028	−0.057 to 0.000	0.138
	Synergy - Sensar	−0.032	−0.064 to −0.001	0.138
**CIVA**	Eyhance	0.213	0.183 to 0.243	Overall: 0.206
	Synergy	0.190	0.153 to 0.226	—
	Sensar	0.247	0.198 to 0.296	—
	Eyhance - Synergy	0.023	−0.022 to 0.069	0.494
	Eyhance - Sensar	−0.034	−0.092 to 0.024	0.494
	Synergy - Sensar	−0.058	−0.120 to 0.005	0.216
**CNVA**	Eyhance	0.050	0.016 to 0.084	Overall: 0.201
	Synergy	0.047	0.006 to 0.088	—
	Sensar	0.109	0.052 to 0.165	—
	Eyhance - Synergy	0.003	−0.047 to 0.053	0.907
	Eyhance - Sensar	−0.059	−0.125 to 0.008	0.256
	Synergy - Sensar	−0.062	−0.133 to 0.010	0.256

Values are adjusted marginal means or between-group differences in logMAR with 95% CIs. Linear mixed-effects models accounted for fellow-eye correlation and were adjusted for age and preoperative spherical equivalent; UDVA and CDVA were additionally adjusted for preoperative distance visual acuity. Pairwise differences represent the first minus the second group, and pairwise *p* values were Holm-adjusted. Analyses included 81 eyes from 50 participants. CI, confidence interval; IOL, intraocular lens; UDVA, uncorrected distance visual acuity; UIVA, uncorrected intermediate visual acuity; UNVA, uncorrected near visual acuity; CDVA, corrected distance visual acuity; CIVA, corrected intermediate visual acuity; CNVA, corrected near visual acuity; logMAR, logarithm of the minimum angle of resolution.

**Table 3:** Adjusted Distance and Near Contrast Sensitivity and Pairwise Comparisons Among the Three Intraocular Lens Groups.

Outcome	Estimate	Adjusted Mean or Difference	95% CI	Adjusted *p* Value
**Distance CS**	Eyhance	1.453	1.402 to 1.504	
	Synergy	1.338	1.276 to 1.401	Overall: 0.007
	Sensar	1.486	1.403 to 1.568	
	Eyhance - Synergy	0.115	0.036 to 0.194	0.01
	Eyhance - Sensar	−0.033	−0.130 to 0.065	0.51
	Synergy - Sensar	−0.147	−0.254 to −0.041	0.01
**Near CS**	Eyhance	1.485	1.429 to 1.541	
	Synergy	1.355	1.287 to 1.422	Overall: 0.01
	Sensar	1.488	1.399 to 1.578	
	Eyhance - Synergy	0.131	0.045 to 0.216	0.008
	Eyhance - Sensar	−0.003	−0.109 to 0.103	0.952
	Synergy - Sensar	−0.134	−0.249 to −0.018	0.04

Values are adjusted marginal means or adjusted between-group differences in log contrast-sensitivity units, with 95% confidence intervals. Estimates were obtained using linear mixed-effects models with IOL type as a fixed effect and a participant-specific random intercept to account for correlation between fellow eyes. Models were adjusted for age and preoperative spherical equivalent. Pairwise differences were calculated as the first-named IOL group minus the second-named group; positive values therefore indicate higher contrast sensitivity in the first-named group. Pairwise *p* values were adjusted for multiple comparisons using the Holm method. The analyses included 81 eyes from 50 participants with complete covariate data. CI, confidence interval; CS, contrast sensitivity; IOL, intraocular lens.

## Data Availability

Deidentified data from the current study are available from the corresponding author upon reasonable request.
